# HOW TO PERFORM LAPAROSCOPIC DISTAL PANCREATECTOMY USING THE CLOCKWISE TECHNIQUE

**DOI:** 10.1590/0102-672020220002e1683

**Published:** 2022-09-16

**Authors:** Adriano Carneiro da COSTA, Duncan SPALDING, Geraldo de Almeida CUNHA-FILHO, Matheus Belem SANTANA, Madhava PAI, Long R JIAO, Nagy HABIB

**Affiliations:** 1 Imperial College London, Department of Hepatobiliary Surgery, Hammersmith Hospital - London, UK.

**Keywords:** Minimally Invasive Surgical Procedures, Laparoscopy, Pancreatectomy, Procedimentos Cirúrgicos Minimamente Invasivos, Laparoscopia, Pancreatectomia

## Abstract

Laparoscopic pancreatectomy is currently a widely used approach for benign and malignant lesions of the pancreas.

**AIMS:**

This study aimed to describe how to perform a laparoscopic distal pancreatectomy using *The Clockwise Technique.*

**METHODS:**

An 18-year-old female patient presented with a well-defined tumor in the pancreatic body with 4 cm in diameter that suggested a diagnosis of solid pseudopapillary tumor (Frantz’s tumor). The patient was recommended for laparoscopic distal pancreatectomy by using *The Clockwise Technique.*

**RESULTS:**

The clockwise, caudal-to-cephalic approach appears to have other significant technical advantages that facilitate the performance of the procedure.

**CONCLUSIONS:**

A laparoscopic distal pancreatectomy performed using *The Clockwise Technique* provides satisfactory outcomes.

## RESUMO - RACIONAL

A pancreatectomia laparoscópica está se tornando uma abordagem amplamente usada para lesões benignas e malignas do pâncreas.

**OBJETIVOS:**

Descrever como realizar a pancreatectomia distal laparoscópica usando a *Clockwise Technique*.

**MÉTODOS:**

Paciente feminina com 18 anos de idade, apresentando tumor bem definido no corpo pancreático com 4 cm de diâmetro que sugeria o diagnóstico de tumor sólido pseudopapilar (tumor de Frantz). O paciente foi considerado para pancreatectomia distal laparoscópica pela *Clockwise Technique*.

**RESULTADOS:**

A *Clockwise Technique* parece apresentar outras vantagens técnicas significativas que facilitam a realização do procedimento.

**CONCLUSÕES:**

A pancreatectomia distal laparoscópica foi realizada com a clockwise technique, obtendo-se resultados satisfatórios.

## INTRODUCTION

Minimally invasive surgery is currently a widely used approach for benign and malignant lesions of the pancreas^2^. Laparoscopic distal pancreatectomy (LDP) is a well-accepted approach to left-sided pancreatic resections and has proven advantages compared its open counterpart and is becoming more frequently performed around the world^3^.

Recent systematic reviews and meta-analysis of studies comparing LPD and open distal pancreatectomy (ODP) have suggested superior short-term outcomes and benefits for patients, including less intraoperative blood loss, shorter postoperative hospital stay, less morbidity, and reduced time to functional recovery for LPD compared to ODP^5^ ^,^ ^6^ ^,^ ^7^. These reviews were recently demonstrated in a randomized controlled trial^4^. However, the oncological safety in terms of resection margins, adequate lymphadenectomy, and survival after LPD in the treatment of pancreatic ductal adenocarcinoma remains controversial, and there has been necessary randomized trial to confirm the oncological safety^8^.

The objective of this study was to describe how to perform an LDP using *The Clockwise Technique.*

## METHODS

**Figure 1 -:**
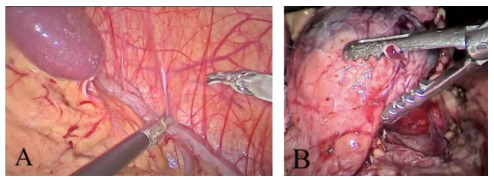
(A) Mobilization of the splenic flexure of the colon. (B) Identification of normal pancreatic neck and visualization of tumor in the body of the pancreas.

An 18-year-old female patient presented with abdominal pain in the upper quadrants. An abdominal ultrasound, a diffusion-weighted imaging, and pancreatic magnetic resonance imaging (MRI) scan showed the presence of a well-defined tumor arising from the pancreatic body with a greater diameter of 4 cm, suggesting the diagnosis of solid pseudopapillary tumor (Frantz’s tumor). The patient was recommended for LDP, using a clockwise technique (Figure 1A and B). Postoperative course was uneventful. Length of hospital stay was 4 days. Final pathology and immunohistochemistry demonstrated a solid pseudopapillary tumor with negative surgical margins.

## RESULTS

**Figure 2 -:**
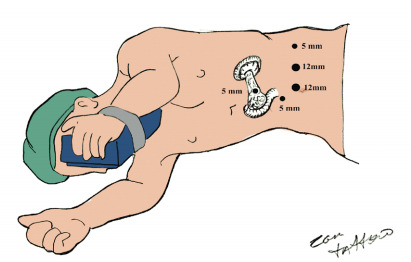
Trocar and patient position in laparoscopic distal pancreatectomy.

The operation is initiated with the patient in a modified right lateral decubitus position that would allow for rotation to the left or right during the procedure. The surgeon stands to the right of the operating table. The ports are placed as shown in Figure 2.

**Figure 3 -:**
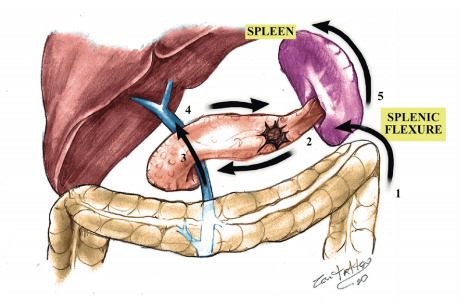
Five steps of *The Clockwise Technique* for laparoscopic distal pancreatectomy.

The clockwise, caudal-to-cephalic approach appears to have other significant technical advantages that facilitate the performance of the procedure. The procedure is performed through the following steps (Figure 3):

- Step 1: Mobilization of the splenic flexure of the colon and exposure of the pancreas.
- Step 2: Dissection along the inferior edge of the pancreas and choosing the site for pancreatic division.
- Step 3: Pancreatic parenchymal division and ligation of the splenic vein and artery.
- Step 4: Dissection along the superior edge of the pancreas.
- Step 5: Mobilization of the spleen and specimen removal.

The total operative time was 300 min with no transfusion. The surgical specimen was removed through a small suprapubic incision, and the abdominal cavity was drained with a closed-suction drain. Recovery was uneventful and patient was discharged on postoperative day 4. Postoperative MRI showed no evidence of the disease 12 months after the procedure.

## DISCUSSION

*The Clockwise Technique* consists of a systematized approach, aiming to facilitate the surgical procedure in a uniform method and well-defined steps^2^ ^,^ ^3^ ^,^ ^6^. This technique appears to offer a reproducible approach to LPD and has been easily assimilated into routine practice.

LPD is a safe and effective method for surgical approach of the pancreas and has significant advantages compared to ODP. The advantages of *The Clockwise Technique* in the laparoscopic approach to pancreatic lesions were initially described in 2011^2^. Asbun et al reported a series of 260 patients who underwent DPL using *The Clockwise Technique* and observed a very low conversion rate (5%), reserved for situations in which the surgeon had difficulty with the anatomy, being mainly early and in the curve of learning^3^. Furthermore, although 87% of resections were related to neoplastic processes, blood loss, transfusion requirements, incidence of pancreatic fistulas, morbidity, and length of hospital stay were lower in LDP compared to ODP. In addition, efficacy of LPD was also observed in lymph node dissection, and long-term survival similar to ODP^2^ ^,^ ^3^.

Abu et al.^1^ described the results of laparoscopic left pancreatosplenectomy exclusively for pancreatic ductal adenocarcinoma performed across two sites in the United Kingdom and the Netherland. Primary outcomes were resection margin and lymph node retrieval. Secondary end points were preoperative outcomes, including postoperative pancreatic fistula. Based on the results in 135 patients evaluated in this study, it was reported that a standardized laparoscopic approach to pancreatic adenocarcinoma in the left pancreas can be adopted safely and the results of this study can be reproduced across multiple sites using the same technique.

## CONCLUSION

LPD was performed using *The Clockwise Technique*, and this systematic approach demonstrated this technique as a reproducible, safe method with low complication rates and, as a consequence, lower morbidity and mortality to the management of lesions on the left side of the pancreas.
